# Dietary supplementation with Lovaza and krill oil shortens the life span of long-lived F1 mice

**DOI:** 10.1007/s11357-014-9659-7

**Published:** 2014-05-10

**Authors:** Stephen R. Spindler, Patricia L. Mote, James M. Flegal

**Affiliations:** 1 Department of Biochemistry, University of California at Riverside, Riverside, CA 92521 USA; 2 Department of Statistics, University of California at Riverside, Riverside, CA 92521 USA

**Keywords:** ω-3 Fatty acids, Lovaza, Krill oil, Marine oil, Mice, Life span, Longevity

## Abstract

Marine oils rich in ω-3 polyunsaturated fatty acids have been recommended as a preventive treatment for patients at risk for cardiovascular diseases. These oils also are the third most consumed dietary supplement in the USA. However, evidence for their health benefits is equivocal. We tested the daily, isocaloric administration of krill oil (1.17 g oil/kg diet) and Lovaza (Omacor; 4.40 g/kg diet), a pharmaceutical grade fish oil, beginning at 12 months of age, on the life span and mortality-related pathologies of long-lived, male, B6C3F1 mice. The oils were incorporated into the chemically defined American Institute of Nutrition (AIN)-93 M diet. An equivalent volume of soybean oil was removed. Krill oil was 3 % and Lovaza 11 % of the oil in the diets. When their effects were analyzed together, the marine oils significantly shortened life span by 6.6 % (*P* = 0.0321; log-rank test) relative to controls. Individually, Lovaza and krill oil non-significantly shortened median life span by 9.8 and 4.7 %, respectively. Lovaza increased the number of enlarged seminal vesicles (7.1-fold). Lovaza and krill oil significantly increased lung tumors (4.1- and 8.2-fold) and hemorrhagic diathesis (3.9- and 3.1-fold). Analysis of serum from treated mice found that Lovaza slightly increased blood urea nitrogen, while krill oil modestly increased bilirubin, triglycerides, and blood glucose levels. Taken together, the results do not support the idea that the consumption of isolated ω-3 fatty acid-rich oils will increase the life span or health of initially healthy individuals.

## Introduction

Isolated marine oils such as fish oil and krill oil are the third most popular dietary supplement in the USA (Hersher 2012). They are thought to produce positive health outcomes because they contain high concentrations of two major n-3-long-chain, polyunsaturated fatty acids (PUFAs; ω-3 fatty acids): eicosapentaenoic acid (EPA; 20:5n-3) and docosahexaenoic acid (DHA; 22:6n-3) (reviewed in Kar 2011; Rizos et al. 2012). Elevated dietary intakes of EPA and DHA can reduce hyperlipidemia in mice (Ruzickova et al. 2004), rats (Rustan et al. 1992), and humans (Mozaffarian and Wu 2012). Marine oils may reduce triglyceride levels, arrhythmias, platelet aggregation, and blood pressure (reviewed in Kar 2011; Rizos et al. 2012). One recent meta-analysis suggested that the consumption of fish and marine n-3 polyunsaturated fatty acids may reduce the risk of breast cancer (Zheng et al. 2013), while another meta-analysis found no change in overall mortality with omega-3 fatty acid consumption (Zhang et al. 2014). In 2004, a concentrated and highly purified fish oil, trade named Lovaza in the USA and Omacor in Europe (Kar 2011), was approved as a drug by the US Food and Drug Administration for the treatment of hyperlipidemia. A number of European regulatory agencies also approved the use of marine PUFAs for reducing cardiovascular risk factors (Rizos et al. 2012).

Despite these approvals and the positive effects on some cardiovascular risk factors, randomized clinical trials have found mixed results regarding the benefits of marine PUFAs for the prevention of adverse cardiovascular events (Marchioli et al. 2002; Yokoyama et al. 2007; Tavazzi et al. 2008; Kromhout et al. 2010; ORIGIN Trial Investigators et al. 2012). A recent meta-analysis of 20 studies and 68,680 patients found no association of PUFA supplementation with lower risk of all-cause mortality, cardiac death, sudden death, myocardial infarction, or stroke based on relative or absolute measures of association (Rizos et al. 2012). Consistent with these results, another meta-analysis found insufficient evidence for a secondary preventive effect of ω-3 fatty acid supplements against overall cardiovascular events among patients with a history of cardiovascular disease (Kwak et al. 2012).

The consumption of dietary marine oils may have adverse consequences. A fish oil-containing diet appears to reduce immune responsiveness to *Helicobacter hepaticus* infection, exacerbating a mouse model of inflammatory colitis and increasing colon cancer risk (Woodworth et al. 2010). Increased serum aspartate aminotransferase and alanine aminotransferase activity, which can be evidence of liver toxicity, are adverse reactions reported in clinical studies of Lovaza (GlaxoSmithKline 2013). Hemorrhagic diathesis also is a postmarketing adverse reaction observed with Lovaza (GlaxoSmithKline 2013). Evidence of increased prostate cancer risk among men with high blood concentrations of long-chain ω-3 PUFAs was reported recently (Bassett et al. 2013; Brasky et al. 2013).

Because of the complex and somewhat paradoxical nature of the results summarized above, we investigated the effects of both Lovaza and food grade krill oil on life span and mortality-related pathology, two key health-related outcomes. We utilized long-lived, male F1 hybrid mice supplemented with krill oil or Lovaza beginning at 12 months of age. The effects of the compounds on food consumption, body weight, and mortality-associated outcomes were determined throughout the study period.

## Results

### Life span

**Fig. 1:**
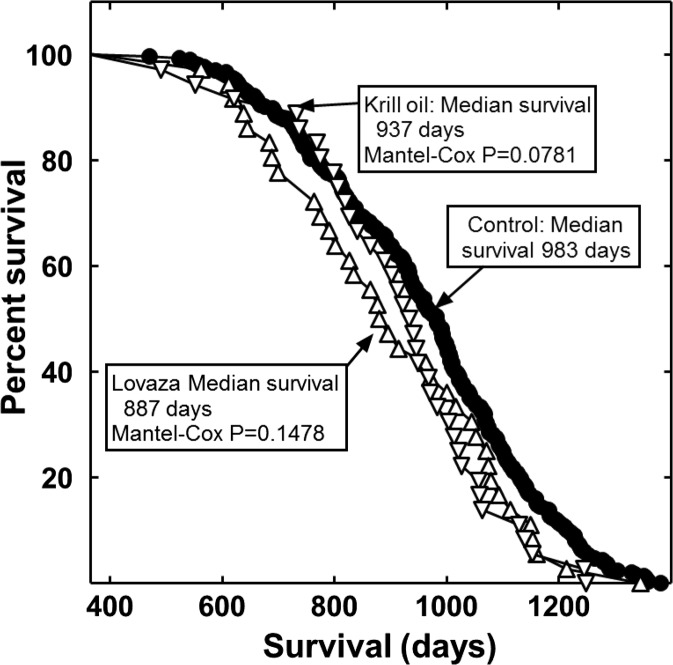
Life span of mice fed a control diet or a diet supplemented with Lovaza or krill oil. Shown are the life spans of the control (*filled circles*), krill oil-treated mice (*downward*-*pointing triangles*), and Lovaza-treated mice (*upward*-*pointing triangles*). The percentage of mice remaining alive at the end of each time period shown is plotted. The controls began with 297 mice and the treatment groups with 36 mice each. The graph begins at 365 days of age, when treatments were started

Male C3B6F1 mice were supplemented beginning at 12 months of age with food containing krill oil or Lovaza. The dosages given and the rationale for them are discussed below. When the effects of the marine oils on longevity were analyzed together using the Mantel–Cox log-rank test, they significantly decreased life span by 6.6 % (*P* = 0.0321). Individually, Lovaza and krill oil non-significantly shortened mean life span by approximately 9.8 and 4.7 %, respectively (Fig. 1). These data probably were not significant individually because of sample size.

These studies were a part of a larger study in which one control group of 297 mice and 58 treatment groups of 36 mice each were used to screen for the effects of different compounds on life span (e.g., Spindler et al. 2013a, b). Other of these treatments increased life span, although most had no effect. For example, treatment of the mice with β-adrenergic receptor blockers or 40 % caloric restriction (CR) produced a significant 8.4 and 23 % increase in life span (Spindler et al. 2013b). In contrast, other compounds produced no effect on life span (e.g., Spindler et al. 2013a, 2014). The sample sizes in this study are similar to those required for a Weibull survival analyses with a 75 % probability of detecting a 10 % increase in mean life span with a 1 % (β ≤ 0.01) probability of a false positive. The Weibull survival analysis is more stringent than the log-rank test (Jeske et al. 2014).

### Rationale for dosages

**Fig. 2:**
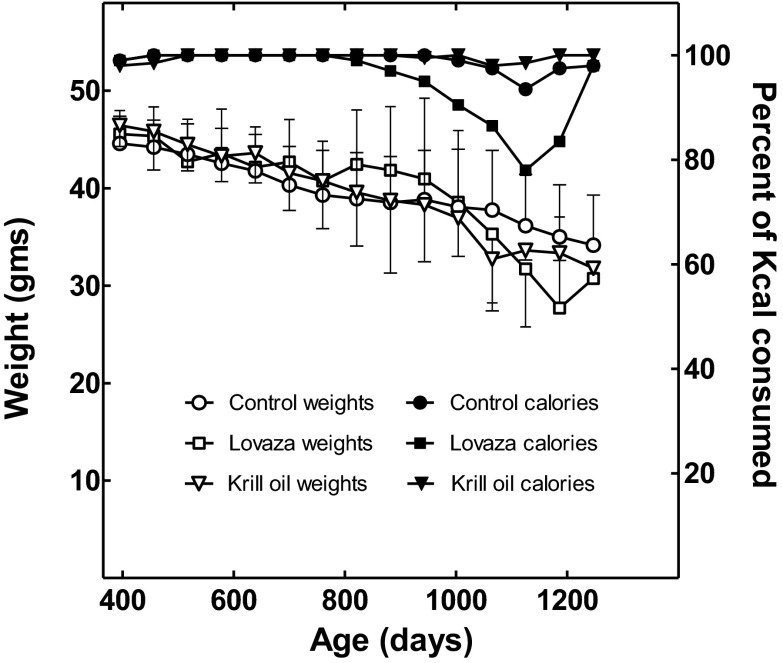
Body weights and food consumption of the mice. The *symbols* are as labeled in the figure. The notation *Percent of Kcal consumed* refers to the percent of the food eaten with respect to the amount presented to the mice in each treatment group per mouse during the preceding 30 days with respect to the amount consumed by the control mice during the same period. For the weights of the mice, the median weight plus or minus the standard deviation is shown. The differences between the weights of the groups were not significant, as judged using a linear mixed effects model as described previously (Spindler et al. 2013a)

Krill oil was administered at 1.17 g oil/kg diet, which is ~124 mg/kg body weight per day (mg/kg bw/day), or approximately 3 % of the oil in the diet. The calculation assumes a body weight of 39 g, which is approximately the median weight of the mice during the treatments (Fig. 2). Lovaza was administered at 4.40 g/kg diet, which is ~467 mg/kg bw/day, or approximately 11 % of the oil in the diet. An equivalent volume of soybean oil was removed from each diet to compensate calorically for the additions. The American Institute of Nutrition (AIN)-93 M diet contains 40 g soybean oil per kg diet, which provides only small amounts of EPA or DHA from the inefficient metabolic conversion of other fatty acids in the oil (Reeves et al. 1993; De Caterina 2011). The krill oil brand used is 23 % EPA and 6.5 % DHA by weight, while Lovaza is 55 % EPA and 45 % DHA by weight. We could not find a literature describing the use of Lovaza in mice. The recommended dose in humans is 4.0 g/day, which would provide 50 mg/kg bw/day for an 80-kg adult. The cross-species scaling factors used to adjust dosages between animals and humans suggest that mice should receive 8 to 12 times the effective human dosage of a drug to account for species-specific pharmacodynamic and pharmacokinetic differences (reviewed in Spindler 2012). The Lovaza dose used here is approximately 9.3 times the recommended human dosage per kilogram body weight. The krill oil dosage is modest in comparison to the dosages used in mouse studies to demonstrate a beneficial effect on serum and hepatic cholesterol and triglyceride levels (Vigerust et al. 2012; Tandy et al. 2009). For example, tumor necrosis factor alpha-transgenic mice fed a high-fat diet containing 5.8 % krill oil (approximately five times the dosage used here) had lower plasma levels of triacylglycerol and cholesterol and higher levels of hepatic mitochondrial and peroxisomal fatty acid β-oxidation and carnitine turnover (Vigerust et al. 2012). High fat-fed mice receiving krill oil at 12.5 g/kg diet (approximately 10 times that used here) had reduced hepatomegaly, hepatic steatosis, triacylglycerols, and cholesterol (Tandy et al. 2009).

### Food consumption and body weight

To determine the possible effects of the compounds on food intake and body weight, the mice were fed a known number of calories each day, and both parameters were quantified throughout the study (Fig. 2). As is typical of meal-fed B6C3F1 mice, all the mice began to lose weight beginning at about 12 months of age, even though they were eating near ad libitum levels of calories (e.g., Spindler et al. 2013a, b; Martin-Montalvo et al. 2013). As in our previous studies, the mean weight estimates became increasingly unstable as days on diet increased, because the number of mice diminished with time (Spindler et al. 2013a, b). Thus, neither the Lovaza nor the krill oil diet produced a consistent treatment effect on body weight (Fig. 2). Anomalously, at 1,125 days of age, the Lovaza diet-fed mice consumed only 78 % of the food offered (Fig. 2). However, only five of the mice remained at this time. Three of these mice died in the ensuing month, and the remaining mice consumed a more “normal” amount of food.

### Necropsies

Necropsy results are shown in Tables 1 and 2. The necropsied control mice were randomly chosen by age at death to approximate the age distribution of the entire cohort. Both Lovaza and krill oil consumption significantly increased the number of mice with peritoneal hemorrhagic diathesis (bleeding into the peritoneum; Table 1). The increase was 3.9-fold for Lovaza and 3.1-fold for krill oil. As mentioned in the “Introduction,” hemorrhagic diathesis is a postmarketing adverse reaction which has been reported for Lovaza by GlaxoSmithKline (2013). We were unable to determine the frequency or severity of this outcome from the information available. In our study, Lovaza treatment also produced a significant increase in the number of enlarged seminal vesicles (7.1-fold; Table 1). There was a 52 % reduction in the median liver tumor mass in the Lovaza-treated mice (Table 2). This size reduction may not have been the result of their shortened life spans, since a similar effect was not found for the krill oil-treated mice (e.g., Dhahbi et al. 2004; Spindler 2005). Lovaza and krill oil treatment produced a significant 4.1- and 8.2-fold increase, respectively, in the number of lung tumors (Table 1). Thus, marine oil consumption was associated with an increase in the onset, incidence, or severity of multiple adverse health outcomes.

**Table 1:** Necropsy results from the mouse longevity studies shown in Fig. 1

Organ	Pathology	Diet treatment (*n*)							
		Control (*n* = 36)^a^		Lovaza (*n* = 35)			Krill oil (*n* = 35)		
		No.^b^	%^c^	No.	%	*P* value^d^	No.	%	*P* value
Spleen	Enlarged/tumorous	23	63.9	23	65.7	1.0000	20	57.1	0.6312
Liver	Tumor	11	30.6	17	48.6	0.1488	13	37.1	0.6210
	Enlarged/fatty liver	1	2.8	1	2.9	1.0000	3	8.6	0.3570
	Hemangioma	4	11.1	1	2.9	0.3570	0	0.0	0.1145
Intestinal	Tumor	5	13.9	8	22.9	0.3723	4	11.4	1.0000
Lung	Tumor	2	5.6	8	22.9	*0.0457*	16	45.7	*9.769e-05*
Penis	Necrosed/inflamed	0	0	2	5.7	0.2394	0	0.0	1.0000
Seminal vesicles	Enlarged	1	2.8	7	20.0	*0.0278*	3	8.6	0.3570
Bladder	Distended	6	16.7	5	14.3	1.0000	2	5.7	0.2603
Kidneys	Enlarged/tumorous	3	8.3	0	0.0	0.2394	2	5.7	1.0000
Thymus	Enlarged	1	2.8	3	8.6	0.3570	4	11.4	0.1987
Skin/abdominal cavity	Fibroma	1	2.8	1	2.9	1.0000	2	5.7	0.6142
Peritoneal cavity	Hemorrhage	4	11.1	15	42.9	*0.0032*	12	34.3	*0.0246*

^a^Number of necropsied mice in each treatment group. The necropsied control mice were chosen randomly from among the mice of appropriate ages for approximating the age distribution of the marine oil-treated mice. One mouse from each treatment group was cannibalized and could not be necropsied

^b^Number of necropsied mice in each treatment group with the indicated pathologies

^c^Percent of the necropsied mice in the treatment group with the indicated pathologies

^d^Fisher’s exact test was utilized to investigate the association between the pathologies and treatment groups. The values that were significantly different or near significance are in italics. The *P* values are two-sided

**Table 2:** Liver tumor mass of the mice shown in Fig. 1

	Control (*n* = 36)	Lovaza (*n* = 35)	Krill oil (*n* = 35)
	Mass (g)^a^	Mass (g)	Mass (g)
Median mass of each tumor	0.54	0.28 (*P* = 0.033)^b^	0.61 (*P* = 0.231)
Tumor mass/number of mice with tumors	1.1	1.1	1.3

^a^One cm^3^ = 1 g

^b^Calculated using the Mann–Whitney *U* test

### Serum tests

To determine whether the decrease in life span was related to drug-induced toxicity, serum samples were analyzed from mice of the same sex and strain fed the Lovaza or krill oil diets (Tables 3 and 4). Most of the parameters measured were not changed significantly by the treatments, suggesting that the oils were not overtly toxic to the liver, muscle, kidney, or other organ systems. Lovaza produced a modest reduction in blood urea nitrogen (Table 3). However, this change was quantitatively modest, not found in the krill oil diet-treated group, and is therefore of uncertain significance. The Lovaza-treated mice did have unkempt hair coats, which can be a symptom of generalized illness.

**Table 3:** Serum tests for Lovaza diet-fed mice. Six treated and six control 19-month-old mice of the same sex and strain used in the life span studies were fed the Lovaza supplemented diet for 16 weeks prior to bleeding by heart puncture

Test^a^	Control	Lovaza	*P* value^b^
Alanine aminotransferase (U/L)	30.3 ± 11.9	28.1 ± 7.5	0.720
Aspartate aminotransferase (U/L)	157.9 ± 36.3	112.4 ± 44.3	0.083
Alkaline phosphatase (U/L)	60.0 ± 10.3	56.3 ± 14.3	0.624
Blood urea nitrogen (mg/dL)	19.1 ± 3.1	14.5 ± 2.7	0.021
Cholesterol (mg/dL)	182.5 ± 21.1	163.9 ± 31.5	0.263
Creatinine (mg/dL)	0.069 ± 0.012	0.076 ± 0.023	0.535
High-density lipoprotein (mg/dL)	196.4 ± 27.3	173.0 ± 34.8	0.229
Low-density lipoprotein (mg/dL)	19.17 ± 3.86	16.74 ± 5.98	0.428
Total bilirubin (mg/dL)	0.093 ± 0.025	0.123 ± 0.045	0.188
Total protein (g/dL)	6.27 ± 0.38	5.94 ± 0.50	0.234
Triglyceride (mg/dL)	69.5 ± 37.0	112.1 ± 99.2	0.362
Glucose, non-fasting (mg/dL)	176.3 ± 24.1	151.3 ± 26.2	0.120

^a^Blood glucose levels were measured with the FreeStyle Lite Blood Glucose Monitoring System (Abbot Laboratories). The other blood tests were performed by the Comparative Pathology Laboratory, University of California, Davis

^b^Calculated using two-sample *t* tests

**Table 4:** Serum tests for krill oil diet-fed mice. Six treated and six control 16-month-old mice of the same sex and strain used in the life span studies were fed the krill oil supplemented diet for 11 weeks prior to bleeding by heart puncture

Test^a^	Control	Krill oil	*P* value^b^
Alanine aminotransferase (U/L)	45.6 ± 29.8	29.3 ± 7.7	0.251
Aspartate aminotransferase (U/L)	106.8 ± 31.2	110.7 ± 17.7	0.798
Alkaline phosphatase (U/L)	73.1 ± 15.2	72.9 ± 9.1	0.982
Blood urea nitrogen (mg/dL)	18.7 ± 2.4	19.9 ± 2.4	0.408
Cholesterol (mg/dL)	186.4 ± 43.4	187.7 ± 34.7	0.957
Creatinine (mg/dL)	0.052 ± 0.017	0.055 ± 0.017	0.817
High-density lipoprotein (mg/dL)	175.9 ± 39.4	179.8 ± 34.4	0.860
Low-density lipoprotein (mg/dL)	28.1 ± 14.0	27.8 ± 10.9	0.963
Total bilirubin (mg/dL)	0.081 ± 0.011	0.115 ± 0.029	0.034
Total protein (g/dL)	6.40 ± 0.63	6.44 ± 0.73	0.921
Triglyceride (mg/dL)	72.0 ± 14.8	95.9 ± 14.8	0.021
Glucose, non-fasting (mg/dL)	111.3 ± 33.9	158.0 ± 23.5	0.024

^a^Blood glucose levels were measured with the FreeStyle Lite Blood Glucose Monitoring System (Abbot Laboratories). The other blood tests were performed by the Comparative Pathology Laboratory, University of California, Davis

^b^Calculated using two-sample *t* tests

The krill oil-treated group showed limited signs of toxicity. Their bilirubin, triglyceride, and glucose levels were modestly elevated (Table 4). Elevation of these parameters can be a sign of liver dysfunction. The Lovaza-treated mice also had numerically elevated triglycerides, although this change did not reach significance. High fat-fed mice receiving krill oil at 12.5 g/kg diet (approximately 10 times that used here) had reduced triacylglycerol and cholesterol levels (Tandy et al. 2009). However, we found an opposite effect on triglycerides for mice consuming the AIN-recommended levels of fat and carbohydrate (AIN-93 M diet; Table 3) (Reeves et al. 1993).

## Discussion

The work presented here shows for the first time that the consumption of either commercial or pharmaceutical grade marine oils can have adverse health effects. The krill oil dosage we used is below that commonly used for the treatment of mice in the scientific literature. In the case of Lovaza, the recommended human dose was scaled up according to accepted cross-species scaling factors. Both oils were present as a relatively small fraction of the total oil in the diet.

Of the pathologies found upon necropsy, hemorrhagic diathesis seems likely to be responsible for a significant proportion of the early mortality of the treated mice. It was substantially and significantly elevated in both the Lovaza- and krill oil-treated mice (Table 1). It is a probable consequence of the anticoagulant effects of dietary marine oils observed in both rodents and humans (Calder and Yaqoob 2012; Sano et al. 2003). The increase in lung tumors in the Lovaza- and krill oil-treated mice also may have contributed to their early mortality. Recent evidence suggests that a diet containing fish oil promotes tumor growth by suppressing CD8^+^ activation in mice (Xia et al. 2014). Thus, the anti-inflammatory effects of marine oils may have negative as well as positive consequences. A fish oil-containing diet significantly decreased the number of CD8^+^ T cells in the lungs of influenza virus-infected mice, leading to elevated rates of morbidity and mortality (Schwerbrock et al. 2009).

Serum tests found little evidence for a significant level of toxicity related to marine oil consumption. The increase in total bilirubin, triglycerides, and glucose in the krill oil-treated group and blood urea nitrogen in the Lovaza-treated mice is modest and not likely sources of early mortality. Thus, there is little evidence that the detrimental effects of these PUFAs on survival are the result of direct toxic effects of the oils or contamination of the oils by the environmental pollutants commonly associated with marine fish, such as methyl mercury, dioxins, or polychlorinated biphenyls (Turunen et al. 2010). Lovaza is a highly purified prescription drug, with very low levels of these toxicants (GlaxoSmithKline 2013).

It is unlikely that the negative effects of these diets on life span were related to the presence of high levels of oxidation products in the oils themselves. The oils were supplied in sealed capsules. These were rapidly opened, combined, and stored sealed in bottles under argon. The bottles were sent to BioServ via expedited shipping. At BioServ, the oils were opened and compounded with the diets, cold pressed into food pellets, and packed in airtight plastic bags. After receipt in the laboratory, the plastic bags of food were stored at −20 °C. Approximately 1-week aliquot of food was transferred from these bags into airtight Rubbermaid tubs, which were stored at −20 °C. The plastic tubs were warmed to room temperature each day before opening for feeding and then returned to the freezer.

As discussed in the “Introduction,” the evidence for an association between marine oil consumption and better health or survival is equivocal. To the best of our knowledge, there are no long-term human or mouse survival studies performed with either Lovaza or other marine oils using initially healthy subjects. A study with senescence-accelerated mice found that the long-term oral administration of fish oil decreased life span (Tsuduki et al. 2011). While these results are similar to ours, studies performed in enfeebled mouse models have a limited predictive value for outcomes in healthy subjects (Spindler 2012). For example, among the many compounds reported to extend the life span of enfeebled mice, only one has been shown to extend the life span of healthy mice (Martin-Montalvo et al. 2013; Anisimov et al. 2005a, b).

F1 hybrid mice were utilized for these studies because they are heterozygous for the alleles differing between their parents. They tend to be more robust and longer lived than their inbred parental lines (Spindler and Mote 2007; Spindler 2012). We have shown that their life span is responsive to multiple interventions including CR (Spindler et al. 2013a), β1-adrenergic receptor antagonists (Spindler et al. 2013b), and metformin (Martin-Montalvo et al. 2013), suggesting that they are appropriate models for testing potential longevity therapeutics.

## Conclusions

Doses of krill oil and Lovaza consistent with those recommended for human use decreased the life span of healthy mice. Marine oil consumption increased deaths associated with hemorrhagic diathesis and specific tumor types. In light of the current uncertainties regarding the efficacy of purified marine oils in treating human diseases, our results do not support their use to increase health or life span.

## Materials and methods

### Mouse husbandry and life span studies

The study design and husbandry conditions are described in detail elsewhere (Spindler et al. 2013a, b). Briefly, male B6C3F1 mice (Harlan Breeders; Indianapolis) were randomly assigned to treatment groups at 12 months of age and switched to daily feeding with 13.3 kcal/day/mouse of control diet (AIN-93 M, Diet No. F05312; BioServ, Frenchtown, NJ). The control group was composed of 297 mice. Test cohorts of 36 mice were fed control diet supplemented with either krill oil (Neptune Krill Oil, Jarrow) or Lovaza (GlaxoSmithKline) at the dosages indicated in the text. Fifty-six other groups of mice were fed other chemical agents in their diets or were calorically restricted. All mice were fed daily. Food consumption was monitored at the time of feeding, and any uneaten food was noted. Dead mice were stored at −20 °C until necropsy. Kaplan–Meier survival curves were compared using the log-rank test implemented in GraphPad Prism 5.01. The probability of detecting a false positive or negative was less than 1 % (Jeske et al. 2014). The statistics were not corrected for multiple testing. The data were not censored. Mice were weighed bimonthly, and the weights recorded. The significance of the differences in weight was determined as described previously (Spindler et al. 2013a). Because the mice were multiply caged, food consumption was determined by totaling the amount eaten by each treatment group during each time period, adjusted for the number of mice in each cage. This number was divided by the amount eaten by the control mice, also adjusted for the number of mice alive per cage.
